# 

**Published:** 1861-02

**Authors:** 


☞ Programme for the Seventeenth Annual Meeting of the Miss. Valley Association of Dental Surgeons:

1. Reports of Officers.
2. Reports of Committees.
3. Election of Officers.
4. Reading of Essays and Papers.
5. Presentation of new Business.
6. Discussion of the following subjects:

    I. Professional Ethics.

II. Structure and Nutrition of Dental Tissues.
III. Reports of Cases— *Written or Verbal.*
IV. Filling Teeth.
V. Mechanical Dentistry—
Continuous Gum,
Vulcanite Base,
Gold Work.

The Executive Committee beg leave to offer the above order of business for the next meeting of the Mississippi Valley Association, Feb. 20th, 1861. Let every member be there, and induce some others to come for the first time.

Respectfully submitted.

G. F. Foote, }

T. F. Davenport, } *Ex. Committee.*

Stoddard Driggs, }

